# α-Amylase Changed the Catalytic Behaviors of Amyloglucosidase Regarding Starch Digestion Both in the Absence and Presence of Tannic Acid

**DOI:** 10.3389/fnut.2022.817039

**Published:** 2022-04-13

**Authors:** Shuangshuang Li, Wenjing Wu, Jing Li, Shengnan Zhu, Xi Yang, Lijun Sun

**Affiliations:** College of Food Science and Engineering, Northwest A&F University, Yangling, China

**Keywords:** α-amylase, amyloglucosidase, starch digestion, competitive inhibitor, binding interactions

## Abstract

The courses of starch digestion with individual α-amylase (AA), amyloglucosidase (AMG), and AA/AMG bi-enzyme system were performed and analyzed by first-order-reaction equations in the absence and presence of tannic acid (TA). An antagonistic effect between AA and AMG occurred at the digestion phase of readily-digestible starch due to the higher catalytic efficiency of AMG for starchy-substrates with more complex structures. This effect caused a faster rate of glucose production with AMG than with AA/AMG bi-enzyme system at this phase both in the absence and presence of TA. TA had a higher binding affinity to AA than to AMG as accessed by several methods, such as inhibition kinetics, fluorescence quenching, isothermal titration calorimetry (ITC), and molecular docking. Besides, differential scanning calorimetry (DSC) indicated that the change in the thermal and structural stabilities of enzymes in the presence of TA was related to the enzyme residues involved in binding with TA, rather than the inhibitory effects of TA. The binding characters of TA to both enzymes resulted in more “free” AMG without TA binding in AA/AMG bi-enzyme system than that in individual AMG. This binding property caused more and faster rate of glucose production at the digestion phase of slowly digestible starch (SDS) in the bi-enzyme system.

## Introduction

Postprandial hyperglycemia is one of the consequences of impaired carbohydrate metabolism (1). Starch is one basic component of main foods for human beings. After ingestion, it is digested by salivary (generally 0–5% starch digested in oral cavity) and pancreatic (most of the starch digested in the small intestine) α-amylase (AA), producing some reducing sugars, such as maltose, maltotriose, maltooligosaccharides, and limited dextrin. These reducing sugars are further catalyzed by brush border α-glucosidases (maltase-glucoamylase and sucrase-isomaltase) of the absorptive enterocytes, producing glucose (2) which is transported into the enterocytes by co-transport with sodium by SGLT1, and then out of the enterocytes into mesenteric blood by the glucose transporter GLUT2 of the basolateral membrane of the enterocytes (3). Therefore, the rate and extent of starch digestion primarily dictate the postprandial blood sugar level (4). Some *in vitro* models of starch digestion in individual AA or AA/amyloglucosidase (AMG) bi-enzyme system have been applied (5, 6), from which the digestion phases and the digestion rate constants in each phase are obtained.

Inhibiting the activity of starch-hydrolyzing enzymes (*e.g*., AA and AMG) has been suggested as a potential approach for controlling starch digestion and reducing the postprandial increase in blood sugar level (7–9). In recent years, phenolic compounds have been widely reported to possess inhibitory activities against AA and thus to delay starch digestion (10, 11). Polyphenol inhibition against AA results from the binding interactions between them (especially for the most common inhibition type, *i.e*., competitive inhibition) that involve amino acid residues of AA and structural moieties of the polyphenol (10, 12, 13). Thus, the inhibitory activity of a polyphenol highly depends on its molecular structure (14, 15). The inhibition and polyphenol-AA binding mechanisms have been characterized by the methods of half inhibition concentration (IC_50_) value, inhibition kinetics, fluorescence quenching, isothermal titration calorimetry (ITC), differential scanning calorimetry (DSC), saturation transfer difference (STD)-NMR, molecular docking, *etc* (10, 16, 17).

In addition, there are several studies focusing on the inhibition effects of polyphenols on α-glucosidase (4, 14) mainly in yeast (such as *Saccharomyces cerevisiae*). However, the standard substrate of yeast α-glucosidase is manufactured as *p*-nitrophenyl-α-_D_-glucopyranoside (*p*NPG), instead of starch naturally-occurring in plants (18, 19). As introduced, AMG has been used along with AA in *in vitro* starch digestion because it plays a similar role as human α-glucosidases. The enzyme AMG can directly hydrolyze starch and further digest the reducing sugars produced by starch hydrolysis catalyzed by AA, and both processes produce glucose as the product (2). Therefore, the inhibition of dietary polyphenols against starch digestion by AMG and AA/AMG bi-enzyme system remains to be characterized. This knowledge would provide a better understanding of the regulating effects of dietary polyphenols on starch digestion and postprandial blood glucose level.

Tannic acid (TA) is a polyphenol composed of 10 galloyl moieties linked with 1 glucosyl (Figure S1). It is a representative tannin compound in grape, persimmon, gallnut, acorn, *etc*. TA has been suggested to be a specific competitive inhibitor of AA (20, 21).

In this study, TA was selected to investigate its influence on starch digestion with the individual enzymes (AA and AMG, respectively) and AA/AMG bi-enzyme system using a first-order reaction analysis. Then, the binding of TA to AA and AMG was characterized using inhibition kinetics, fluorescence quenching, ITC, DSC, and molecular docking methodologies to determine how the interactions inhibited starch digestion differently.

## Materials and Methods

### Materials and Chemicals

Tannic acid, AA (EC 3.2.1.1) from porcine pancreas, AMG (EC 3.2.1.3,) from *Aspergillus niger*, phosphate-buffered saline (PBS) tablets, and *p*-hydroxybenzoic acid hydrazide (PAHBAH) were purchased from Sigma-Aldrich Co. (St. Louis, US). Normal maize starch was obtained from Yuanye Biotech. Co. (Shanghai, China). A _D_-glucose assay kit (GOPOD-FORMAT) was obtained from Megazyme® (Megazyme E-AMGDF). Other chemicals were of analytical grade.

### Starch Digestion in the Absence and Presence of TA

The courses of starch digestion with AA, AMG, or AA/AMG in the absence and presence of TA were carried out according to previous studies (22–24). Specifically, 10 mg/ml of normal maize starch was fully gelatinized at 90°C for 40 min. TA was dissolved in buffer solution with a final concentration of 30 mg/ml. The concentration of AA was 200 U/ml (1U: mM maltose released/min), and the concentration of AMG was 30 U/ml (1U: mM glucose released/min). For the starch digestion by AA/AMG, 0.75 ml of TA was added into a 15 ml tube containing a mixture of 1 ml of AA and 0.25 ml of AMG solutions (with the ratio of AA to AMG used in this study, the rate of starch digestion can be reasonably controlled during 1 h), followed by incubation at 4°C for 10 min to maximize the binding interactions between TA and the enzymes. The control was 0.75 ml of buffer solution mixed with the enzymes.

To initiate the digestion process, 2.5 ml of gelatinized starch was added to the mixture of TA and enzymes, and the reaction tubes were incubated at 37°C with a magnetic stirring speed of 600 rpm. As for starch digestion with the individual enzymes, AA or AMG was replaced by a buffer solution with the equivalent volume, respectively. Then, at each interval during the digestion process, some amount of reaction solution was withdrawn into a tube containing a solution of Na_2_CO_3_ to stop the reaction (for starch digestion by AMG and AA/AMG, 150 μl of reaction solution into 300 μl of 0.3 M Na_2_CO_3_, and for digestion by AA, 100 μl of reaction solution into 1,000 μl of 1 M Na_2_CO_3_). The tubes containing the stopped reaction solution were centrifuged at 7,300 × g force for 10 min, and the supernatants were withdrawn for product analyses as follows.

For the starch digestion with AA, the reducing sugar contents in the supernatants were determined using the PAHBAH method (25). The fraction of digested starch at each interval timepoint was converted using the coefficient of 324/342 (the disaccharide residue molecular mass ratio from maltose to starch). For the starch digestion with AMG or AA/AMG, the glucose contents in the supernatants were determined using the GOPOD method (26). The fraction of digested starch at each interval timepoint was converted using the coefficient of 162/180 (the monomer residue molecular mass ratio from glucose to starch). The relationship between the digested starch fraction and digestion time was analyzed using the first-order reaction equation (1) as follows (5):


(1) $$\begin{array}{l} C_{t}=C_{\infty }\left(1-e^{-kt}\right) \end{array}$$

where *C*_t_ is the fraction of digested starch at digestion time *t*; *C*_∞_ is the fraction of digested starch at digestion termination; *k* is the digestion rate constant; *t* is the digestion time.

To obtain the *k* value, the equation (1) was rewritten into a logarithm of slope (LOS) equation as follows (5):


(2) $$\begin{array}{l} \ln\left(\frac{dC_{t}}{dt}\right)=-kt+\ln\left(C_{\infty }k\right) \end{array}$$

For starch digestion with a single phase, the LOS plot was shown with a linear character. If there exist multiple digestion phases, the piecewise first-order equation (3) was used as follows (5):


(3) $$Ct=\{\begin{array}{c} C_{1}+C_{1\infty }\left(1-e^{-k_{1}t}\right)\text{​},\;0\leq t\leq t_{1}\\ C_{2}+C_{2\infty }\left(1-e^{-k_{2}t}\right)\text{​},\;t_{1}\leq t\leq t_{2}\\ \ldots \\ C_{n}+C_{n\infty }\left(1-e^{-k_{n}t}\right)\text{​},\;t_{n-1}\leq t\leq t_{n} \end{array}$$

where *C*_n_ is the initial fraction of digested starch at each phase; *n* is the phase order; *k*_n_ and *C*_n∞_ are the corresponding digestion rate constant and the fraction of digested starch at the digestion termination of each phase; *t*_n_ is the terminal timepoint of each phase.

### Inhibition of Enzymes and Kinetics

#### Inhibitory Activity of TA Against the Enzymes

The IC_50_ value was used to describe the inhibitory activity of TA against two enzymes, which was determined and calculated according to the inhibition percentages (%) for a series of TA concentrations. In detail, starch digestion procedures were conducted with 4 ml of 10 mg/ml starch, 50 μl of TA and 50 μl of 4 U/ml AA for AA inhibition determination; with 2.5 ml of 10 mg/ml starch, 80 μl of TA and 80 μl of 14 U/ml AMG for AMG inhibition determination. At the digestion interval of 3 min (up to 12 min), 300 μl of AA catalyzing hydrolysate and 150 μl of AMG catalyzing hydrolysate were withdrawn for the determination of reducing sugar and glucose contents according to the PAHBAH and GOPOD methods, respectively. The initial digestion velocity (*v*) was obtained from the slope of the linear relationship between reducing sugar or glucose contents and the digestion time (*t*). Then, the inhibition (*I*) was calculated according to equation (4) as follows:


(4) $$\begin{array}{l} I\;\left(\%\right)=\left(1-\frac{v}{v_{0}}\right)\times 100 \end{array}$$

where *v* and *v*_0_ are the initial digestion velocities of starch digestion in the presence and absence of TA, respectively. Then, the IC_50_ value was calculated using the equation (5) as follows (21, 27):


(5) $$\begin{array}{l} I=I_{\max}\left(1-\frac{IC_{50}}{\left[I\right]+IC_{50}}\right) \end{array}$$

where [*I*] is the inhibitor concentration; *I* is the enzyme inhibition at each inhibitor concentration; *I*_*max*_ is the estimated maximum inhibition.

#### Inhibition Kinetics of TA

Lineweaver–Burk, Dixon, and Cornish–Bowden plots were applied to describe the kinetics of inhibition. The values of initial digestion velocity (*v*) were determined as described above with 4 ml of gelatinized starch (2.5, 5, 10, and 15 mg/ml), 50 μl of TA (0, 10, 20, 30, and 40 mg/ml), and 50 μl of 2.5 U/ml AA for AA inhibition kinetics; with 2.5 ml of gelatinized starch (2.5, 5, and 10 mg/ml), 80 μl of TA (0, 10, 20, 30, 40, and 50 mg/ml), and 80 μl of 11 U/ml AMG for AMG inhibition kinetics. Notably, the concentrations of TA and starch were selected according to the excepted linear characters of the equations applied in the inhibition kinetics analysis.

After obtaining the *v* values for a series of starch and TA concentrations, the competitive inhibition constant (*K*_ic_) and the uncompetitive inhibition constant (*K*_iu_) were obtained from the respective intersection points of Dixon (6) and Cornish–Bowden (7) equations as follows (28, 29):


(6) $$\begin{array}{l} v=\frac{V_{\max}a}{K_{m}\left(1+\frac{i}{K_{ic}}\right)+a} \end{array}$$

(7) $$\begin{array}{l} \frac{v}{a}=\frac{V_{\max}}{K_{m}\left(1+\frac{i}{K_{ic}}\right)+a\left(1+\frac{i}{K_{iu}}\right)} \end{array}$$

Besides, the maximum reaction velocity (*v*_max_) and the Michalis constant (*K*_m_) were obtained according to the intercept (1/*v*_max_) and slope (*K*_m_/*v*_max_) of Lineweaver–Burk equation (8) as follows (30):


(8) $$\begin{array}{l} \frac{1}{\text{v}}=\frac{1}{{\text{v}}_{\max}}+\frac{{\text{K}}_{\text{m}}}{{\text{v}}_{\max}}\frac{1}{\text{a}} \end{array}$$

where, for the kinetics equations above, *i* and a are the concentrations of TA and starch, respectively.

### Binding Interactions Between TA and Enzymes

#### Fluorescence Quenching

The fluorescence spectra of AA and AMG in the absence and presence of TA were determined according to previous studies (31, 32). For the fluorescence quenching effect on AA, 30 μl of TA (0.0625–1.0 mg/ml) and 400 μl of 0.117 mg/ml AA were applied. For the fluorescence quenching effect on AMG, 30 μl of TA (0.0625–1.0 mg/ml) and 400 μl of 0.8 mg/ml AMG were used. The fluorescence quenching constant (*K*_FQ_) was obtained from the Stern-Volmer equation (9) as follows (31, 32):


(9) $$\begin{array}{l} \frac{F_{0}}{F}=1+k_{q}\tau_{0}\left[Q\right]=1+K_{FQ}\left[Q\right] \end{array}$$

where *F*_0_ and *F* are the fluorescence intensities in the absence and presence of TA, respectively; *k*_q_ is the bimolecular quenching constant; τ_0_ is the lifetime of the fluorophore, and for AA this value is 2.97 ns, and for AMG it is 10^−8^ s (31–33); [*Q*] is the TA concentration.

In the case that there were both static and dynamic mechanisms or a “sphere-of-action” (apparent static mechanism), the modified exponential Stern–Volmer equation (10) was applied to obtain *K*_FQ_ as follows (31, 32):


(10) $$\begin{array}{l} \frac{F_{0}}{F}=e^{\left(K_{FQ}\left[Q\right]\right)} \end{array}$$

#### Isothermal Titration Calorimetry

A TA® NanoITC isothermal titration calorimeter instrument was used to detect the enthalpy changes caused by the binding interactions between TA and the enzymes (17, 34). Specifically, 50 μl of 5 mg/ml TA was loaded into an ITC syringe and was titrated into an ITC sample cell containing 170 μl of 1.5 mg/ml AA (or 7.5 mg/ml AMG) solution. The number of total injections was 25 with the volume of each injection of 2 μl. The duration of each injection was 180 s. The temperature during the titration process was maintained at 25°C with a stirring speed of 250 rpm in the sample cell. The control was set as the titration of TA to the buffer solution. The raw data of ITC were obtained as a plot of heat rate (μJ/s) against time (s). Then, the data were integrated peak-by-peak and normalized to obtain a plot of corrected enthalpy per molar injection (kJ/mol) against the injection order. The plot was then fitted using an independent (single-site) binding model within the modified and available ranges of molar ratios of TA to enzymes to ensure a better fit of the model and the related constants calculated. The equation (11) of this binding model is suggested as follows (17):


(11) $$\begin{array}{l} Q_{i}=\frac{n\left[M\right]HV_{0}}{2}\left\{1+\frac{\left[P\right]}{n\left[M\right]}+\frac{K_{d}}{n\left[M\right]}\right\}\\ -\left\{\sqrt{{\left(1+\frac{\left[P\right]}{n\left[M\right]}+\frac{K_{d}}{n\left[M\right]}\right)}^{2}-4\frac{\left[P\right]}{n\left[M\right]}}\right\} \end{array}$$

where *Q*_*i*_ is the total heat collected after the injection *i*; *V*_0_ is the volume of the ITC sample cell (170 μl); [*M*] is the total concentration of enzymes; [*P*] is the total concentration of TA; *n* is the molar ratios of interacting molecules (stoichiometry); Δ*H* is the enthalpy changes; *K*_*d*_ is the equilibrium dissociation constant of TA-enzyme complex.

#### Differential Scanning Calorimetry

The thermal stability of enzymes in the absence and presence of TA was determined by use of a TA® Q2000 differential scanning calorimeter (17, 34). Specifically, 50 μl of the respective enzyme solution (~50 mg/ml) was mixed with 50 μl of TA (60 mg/ml), followed by incubation at 4°C for 15 min. Then, 15 μl of the mixture was pipetted into a TA® Tzero pan and sealed tightly with a Tzero hermetic lid. A mixture of enzymes with buffer solution was applied as the control. The reference (empty) and sample pans were placed onto the DSC heating platform. The thermograms were collected from 10 to 120°C with a 5°C/min heating rate. Both the denaturation temperature (*T*_d_, °C) and denaturation enthalpy (Δ*H*_dsc_, J/g protein) required to fully denature the enzymes were obtained.

#### Molecular Docking

A Sybyl 2.0 molecular docking software was used to predict the interaction forces and sites of TA with the enzymes. The crystal structures of AA and AMG were extracted from the Protein Data Bank. The binding energy, *E*_b_ was calculated based on the equation (12) as follows (21, 35):


(12) $$\begin{array}{l} E_{b}=RT\log_{e}\left(10^{-pk_{d}}\right) \end{array}$$

where *pk*_d_ is the affinity score according to the Surflex scoring function, and the *RT* is 0.59 kcal/mol.

### Statistical Analysis

One-way analysis of variance (ANOVA) followed by Tukey's test (Graphpad Prism 6) was applied to analyze the significant difference between the selected constants. The data were considered statistically significant at *p* < 0.05 and marked with different superscripts in the figures and tables.

## Results and Discussion

### Inhibition of Starch Digestion by TA

**Figure 1:**
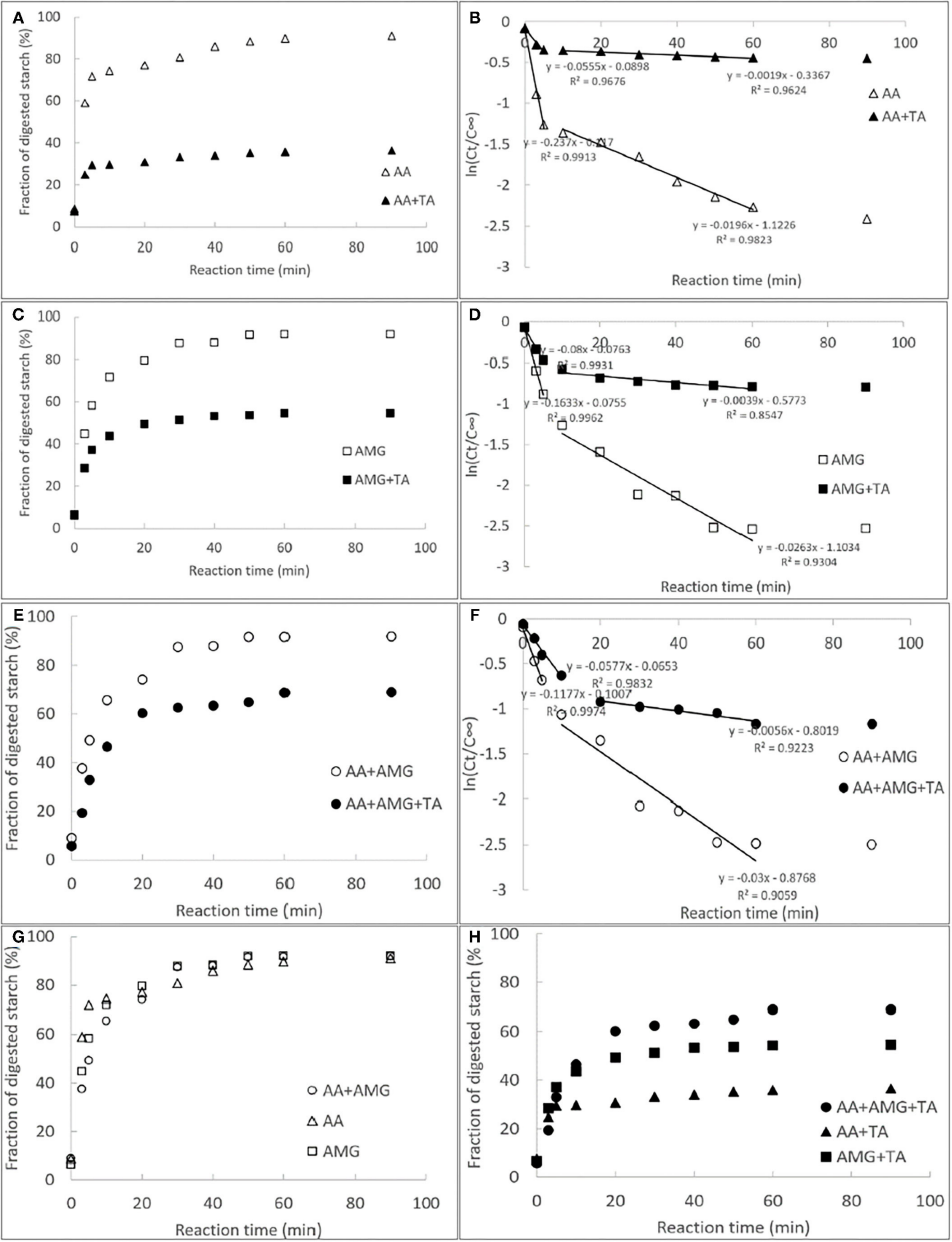
The courses of starch digestion by individual α-amylase (AA) **(A)**, amyloglucosidase (AMG) **(C)** and AA/AMG bi-enzyme system **(E)** in the absence and presence of tannin acid (TA) (30 mg/ml), and the respective fitted first-order logarithm of slope (LOS) plots **(B,D,F)**; in the LOS plots, the slopes of the fitted equations of at each digestion phases were calculated and defined as the digestion rate constants (*k*). The former and latter half in individual digestion profiles were the digestion phases of rapidly digestible starch (RDS) and slowly digestible starch (SDS), respectively. To describe the starch digestion processes in different enzymic systems more clearly, all the courses in the absence **(G)** and presence **(H)** of the polyphenol were specifically summarized.

The courses of starch digestion with AA, AMG, or AA/AMG bi-enzyme system in the absence and presence of TA were performed and analyzed using the first-order reaction equation for multiple digestion phases (Figure 1). The digestion rate constants (*k*) at each phase were also calculated (Figure 1). A higher *k*-value indicates a higher digestion velocity (*v*) and thus a higher catalytic activity of the respective enzymes. Interestingly, although both AMG and AA were able to digest starch substrate, the catalytic velocity of AA was higher than that of AMG (Figures 1B,D). This finding may result from the fact that AMG digests starch molecules from the non-reducing terminals, producing glucose (2, 24), while AA digests starch at any positions linked with α-1,4-glucosidic bonds, producing reducing sugars (maltose, maltotriose, maltooligosaccharides, *etc*.) (2). Besides, the starch digestion velocity with AA/AMG bi-enzyme system was slower than that with individual AMG for the digestion phase of rapidly digestible starch (RDS) (before 5 min, Figures 1D,F,G), both of which were characterized by the amount of glucose produced. This finding is in accordance with previous studies in which AA and AMG have been reported to show an antagonistic effect on the digestion of gelatinized starch (2, 24). This result can be explained by the difference in the efficiency of starch hydrolysis between AA and AMG. AA efficiently hydrolyzes starch to low-molecular-weight maltooligosaccharides. AMG hydrolyzes polymeric starch and maltooligosaccharides with longer skeletons more efficiently than oligomeric saccharides.

It should be noted that a lower ratio of *k* (digestion rate constant) in the presence of TA to that in the absence of TA suggests a higher inhibition effect of TA on starch digestion catalyzed by the enzymes. Therefore, the strongest inhibitory effect of TA was observed on starch digestion by AA among the three enzymes (Figures 1B,D,F). The antagonistic effect between AA and AMG caused a faster rate of glucose production with AMG than with AA/AMG bi-enzyme system at RDS digestion phase in the presence of TA (Figures 1D,F). However, at slowly digestible starch (SDS) digestion phase where there was no antagonistic effect between the two enzymes, more and faster rate of glucose production in the presence of TA was found with AA/AMG bi-enzyme system than with AMG (Figures 1D,F,H). This result may arise from the fact that TA was able to bind with both AA and AMG. Therefore, there were more “free” AMG without TA binding in AA/AMG bi-enzyme system than in the individual AMG.

To further elucidate the inhibiting behaviors of TA on starch digestion with different enzymes, the details of enzyme inhibition and TA-enzyme binding interactions are discussed as follows.

### Enzyme Inhibition and Kinetics of TA

**Figure 2:**
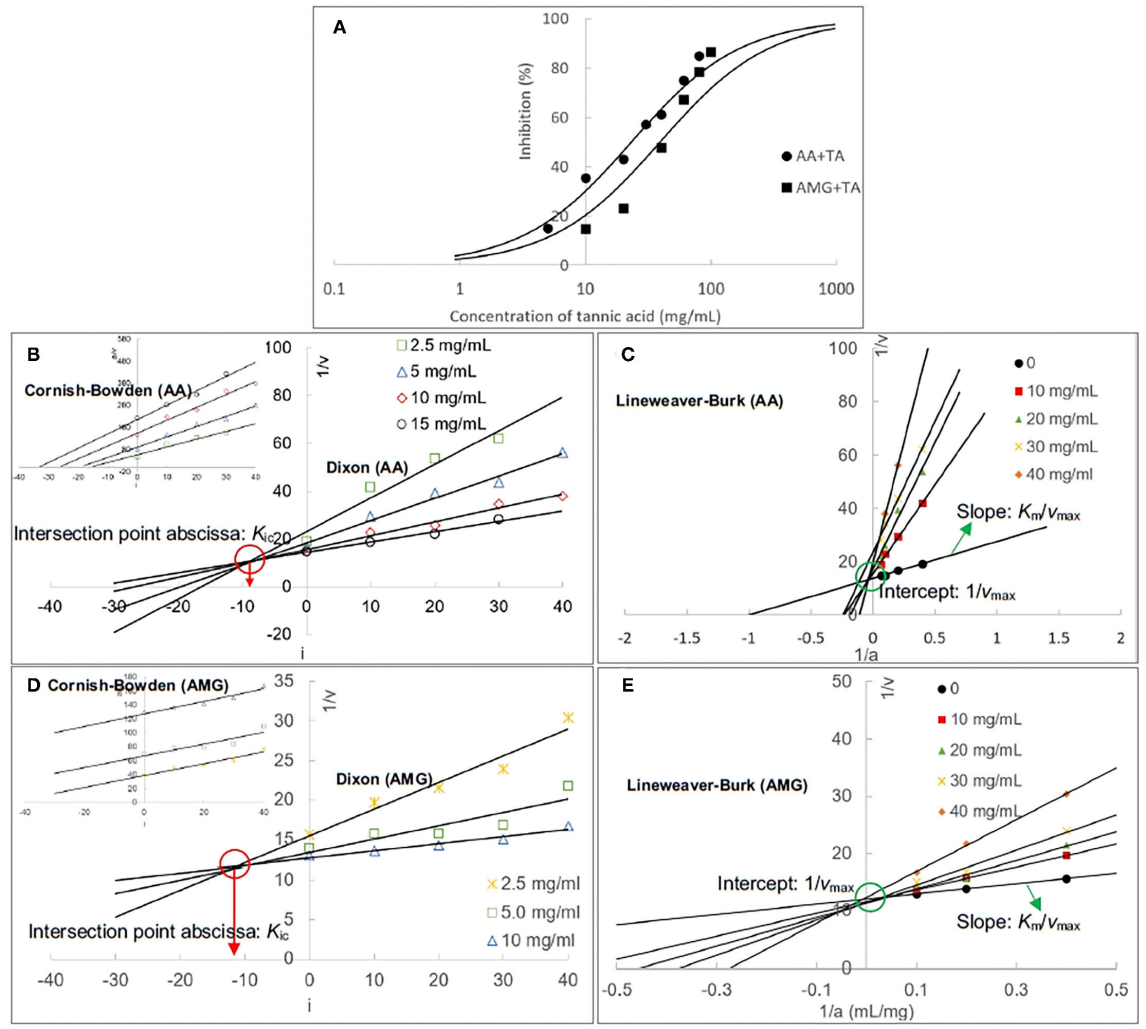
The inhibition of AA and AMG at different TA concentrations **(A)**; the inhibition curves were fitted using the half inhibition concentration (IC_50_) calculating equation. The Dixon and Cornish–Bowden (insert) plots of TA for AA inhibition **(B)**, and the respective ones for AMG inhibition **(D)**, in which the absolute value of intersection abscissa in the Dixon plot was calculated as the competitive inhibition constant (*K*_ic_). The Lineweaver–Burk plots for AA **(C)** and AMG **(E)** inhibition, in which the intercept was calculated as the reciprocal of maximum initial reaction velocity (1/*v*_max_) and the slope was the ratio of Michaelis constant to *v*_max_ (*K*_m_/*v*_max_).

The inhibitory activity of TA against AA and AMG was characterized by the IC_50_ values that were calculated according to the enzyme inhibition at a series of TA concentrations (Figure 2A). It was found that the inhibitory activity of TA against AA was higher than that against AMG because the IC_50_ value for AA inhibition was lower (Table 1), resulting in the higher inhibition of starch digestion with AA than with AMG (Figures 1B,D).

**Table 1:** The constants that indicate the enzyme inhibition of tannin acid (TA) and the binding interactions between TA and two enzymes.

**Enzymes**	**IC_**50**_ (mg/ml)**	**Inhibition type**	***K*_ic_ (mg/ml)**	**1/*K*_ic_ (ml/mg)**	***K*_FQ_ (L/mol)**	***k*_q_ [L/(mol•s)]**	***K*_d_ (mol/L)**	***K*_itc_ (L/mol)**	**Δ*H*_itc_ (kJ/mol)**	**Δ*H*_dsc_ (J/g)**	***T*_d_ (**°**C)**	***E*_b_ (kcal/mol)**
AA	23.16^b^	Competitive	8.49^b^	0.118^a^	3.42 × 10^3a^	1.15 × 10^12a^	2.54 × 10^−6*b*^	3.94 × 10^5a^	−11.46^a^	3.54^A^ (3.99^A^ for control)	92.64^A^ (92.54^A^ for control)	−5.21^b^
AMG	39.16^a^	Competitive	11.14^a^	0.090^b^	2.27 × 10^2b^	2.27 × 10^11b^	2.70 × 10^−5*a*^	3.70 × 10^4b^	−5.218^b^	0.365^B^ (0.550^A^ for control)	73.09^B^ (76.49^A^ for control)	−7.37^a^

*The different superscript letters in the same column indicate the significantly different (p < 0.05) with each other. The letters labeled for the thermal stability (ΔH_dsc_ and T_d_) in the same line indicate the significant difference with the respective control*.

Then, the inhibition kinetics of AA and AMG by TA were studied with the Dixon, Cornish–Bowden, and Lineweaver–Burk equations to obtain the inhibition type and inhibition constants (Figures 2B–E). It was found that both the Dixon plots of AA and AMG inhibition by TA intersected at one point, while the Cornish–Bowden plots showed that they were paralleled to each other (Figures 2B,D), indicating that TA was a competitive inhibitor of AA and AMG. Therefore, it can bind with the respective active sites of the two enzymes, competing for the starch substrate (29, 36). As the 1/*K*_ic_ value for AA inhibition was higher than that for AMG inhibition (Table 1), TA had a higher binding affinity to AA than to AMG at the active sites, which caused the stronger inhibitory activity of TA against AA (Figure 2A, Table 1).

Supportively, the equation lines in the respective Lineweaver–Burk plots of AA and AMG inhibition intersected at one point that was close to *y*-axis (Figures 2C,E). Taking the experiment errors and equation application deviations (especially at a relatively high inhibitor concentration) into accounts, the intersection point in the Lineweaver–Burk plot of the enzyme inhibition was considered as the intercept of the kinetic equation that is defined as the reciprocal of maximum reaction velocity (1/*v*_max_) (Figures 2C,E). The 1/*v*_max_ values at different TA concentrations were stable for both AA and AMG inhibition (Figures 2C,E). Besides, the slopes of Lineweaver–Burk plots that are defined as the ratio of Michaelis constant to *v*_max_ (*K*_m_/*v*_max_) increased with the increasing TA concentration (Figures 2C,E), suggesting that the *K*_m_ value was increased due to the inhibition of AA and AMG by TA.

Therefore, by combining *v*_max_ and *K*_m_ parameters, the Lineweaver–Burk kinetics analysis showed that TA competitively inhibited both enzymes (30), which confirmed the result of Dixon and Cornish–Bowden equation analysis.

### Characterization of Binding Interactions Between TA and Enzymes

#### Fluorescence Quenching

There are some aromatic amino acid residues in AA and AMG, such as tryptophan and tyrosine, which provide the enzymes with specific fluorescent properties at certain excitation wavelengths of ultraviolet light (21). When there exist exogenous quenchers that have aromatic rings (*e.g*., benzene rings of polyphenols), non-covalent π-π conjugations (stackings) between the aromatic rings of polyphenols and that of the enzymes would form. The non-covalent interactions are able to “cover” the fluorescence property of the enzyme fluorophores (31, 34). Thus, the fluorescence quenching effects of polyphenols can be observed.

**Figure 3:**
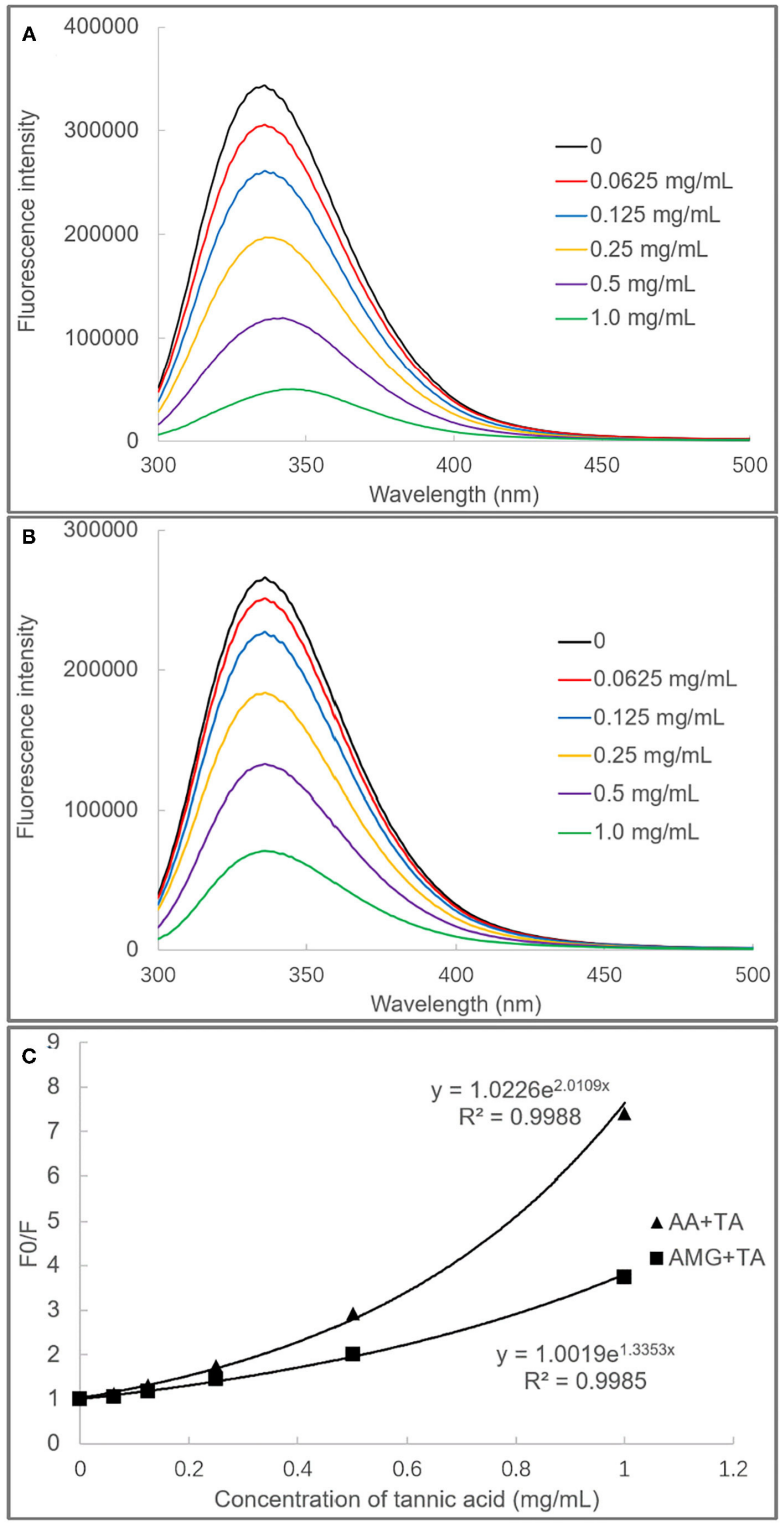
The fluorescence spectra of AA **(A)** and AMG **(B)** in the absence and presence of TA, and the fluorescence quenching effects of TA were analyzed by the modified (exponential) form of Stern–Volmer equation **(C)**.

It was found that TA quenched both the fluorescence of AA and AMG in a TA concentration-dependent mode (Figures 3A,B). The plots of *F*_0_/*F* (the ratio of maximum fluorescence intensity in the absence of TA to that in the presence of TA) against TA concentrations were shown with a character that concaved toward *y*-axis (Figure 3C). This result indicated that TA quenched the enzyme fluorescence by a “sphere-of-action” mechanism (31, 32). To further confirm this mechanism, the bimolecular quenching constants (*k*_q_) of TA were calculated. Notably, the *k*_q_ value for a specific dynamic quenching is approximately 1 × 10^10^ M^−1^s^−1^, and the *k*_q_ values of TA for AA and AMG quenching were 115 and 22 times of this value, respectively (Table 1). These higher values indicated that TA quenched the fluorescence of both enzymes in a static mode, *i.e*., polyphenol-enzyme complexes were formed between TA and AA and between TA and AMG.

Besides, from the exponential Stern–Vomer equation, the fluorescence quenching constants (*K*_FQ_) of TA indicated that the binding interactions of TA with the fluorophores of AA were stronger than with that of AMG (Table 1). Therefore, both fluorescence quenching and inhibition kinetics indicate stronger binding interactions between TA and AA as compared with TA and AMG.

#### Isothermal Titration Calorimetry

**Figure 4:**
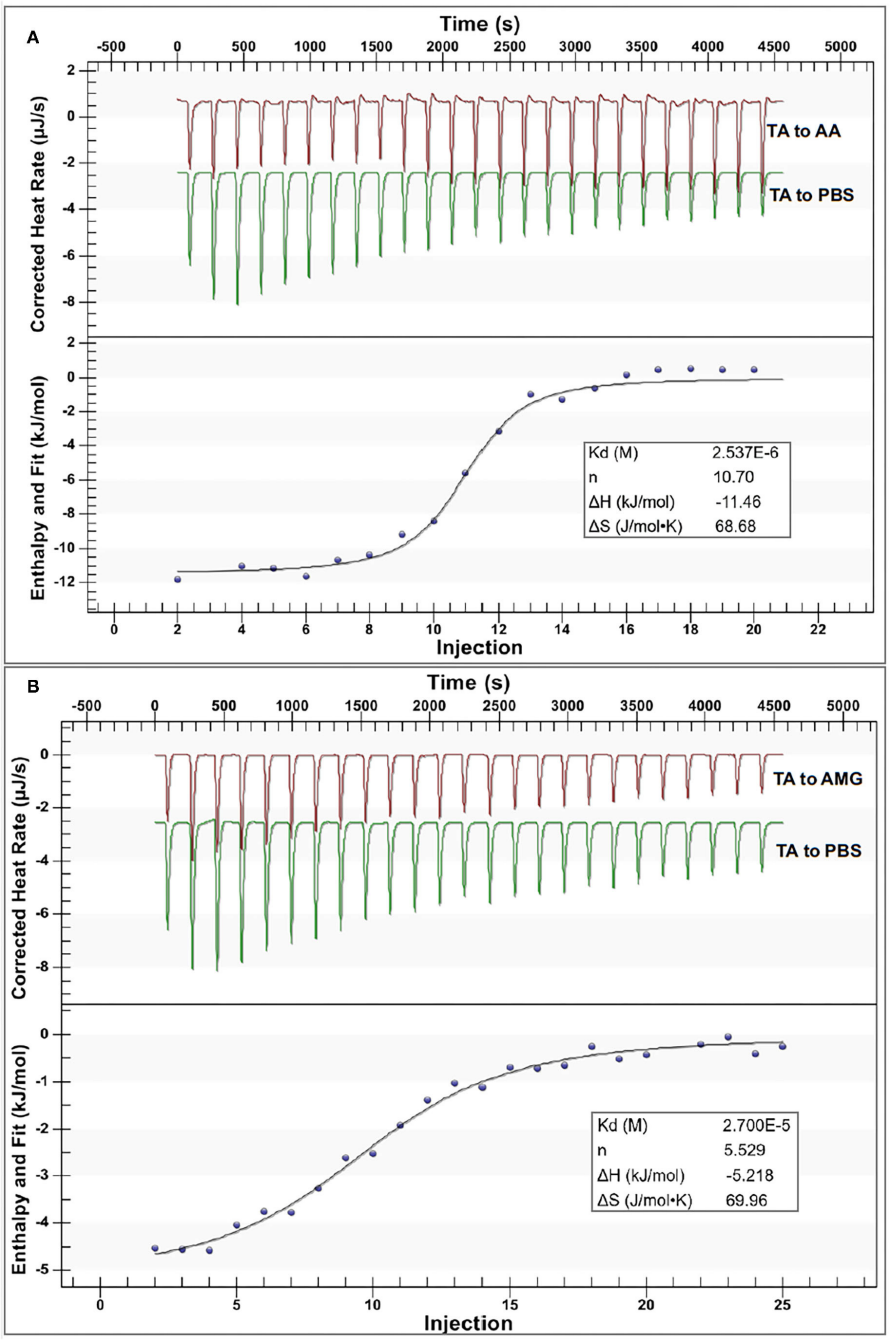
The isothermal titration calorimetry (ITC) data for binding interactions between TA and AA **(A)** and that between TA and AMG **(B)**; in each figure, the upper section indicates the heat flow for titration of TA to the enzyme solution (the red curves) and titration of TA to buffer (the green curves), and the lower section indicates the fitted plots of the enthalpy changes against the injection order by the use of an independent binding model equation. Notably, the applied molar ratios of TA to the enzymes were modified within the available ranges to make the model fit the curves more suitably.

The ITC method was applied to detect and collect the heat flow during the titration of TA to the enzymes (Figure 4). Notably, the heat flow was observed during the titration process of TA to PBS buffer (Figure 4), which was the dilution heat of TA (17). After subtracting the dilution heat of TA from the titration heat of TA to the enzyme solutions, the enthalpy changes (Δ*H*) caused by TA-enzyme binding interactions were obtained (Figure 4). The negative Δ*H* values indicate that both the binding interactions of TA with AA and AMG were an exothermal process, which conforms with the character of enthalpy changes for the binding of a phenolic micromolecule with a protein macromolecule (37). Then, an independent (single-site) binding model was a good fit of the relationships between Δ*H* and injection order (*i.e*., the molar ratio of TA to enzyme) for both the titration processes (Figure 4). This result indicated that the respective binding sites of both enzymes with TA tended to be similar, corresponding to the competitive inhibition characters of TA against both enzymes (Figures 2B–E).

From the independent binding mode equation, the *K*_d_ values that indicate the dissociation constants of the TA-enzyme complexes were obtained (Figure 4). By definition, the binding (association) constants (*K*_itc_) of TA with the respective enzymes were calculated as the reciprocal of *K*_d_ (1/*K*_d_) (27) (Table 1). A higher *K*_itc_ value indicates a higher binding affinity of TA to the enzyme. Therefore, TA had a higher binding affinity to AA than to AMG (Table 1). To support this, a higher Δ*H* value per molar TA titration (Δ*H*_itc_) to AA than to AMG was observed (Table 1), as stronger binding interactions should result in more energy released. Interestingly, this ITC result confirmed the results of fluorescence quenching and inhibition kinetics (Table 1). In addition, the higher binding affinity of TA to AA corresponded to the higher inhibitory activity of TA against AA (Table 1). On the other hand, the stoichiometry (*n*) that indicates the molar ratio of the ligand to the enzyme required to saturate the enzyme binding sites, as well as the entropy changes per molar titration (Δ*S*_itc_) that indicates the degree of the order of the ligand-enzyme complex system, are two constants highly related with the number of binding sites and enzyme molecular conformation (34, 37). Therefore, the difference in the structural properties of enzymes caused the difference in their values of *n* and Δ*S*_itc_ for binding with TA (Figure 4). In addition, the binding parameters of TA to the respective enzymes indicate that TA bound with both enzymes in the bi-enzyme system. This phenomenon caused the different inhibition behaviors of TA on starch digestion in an individual AMG system and in an AA/AMG bi-enzyme system (Figures 1D,F,H).

#### Differential Scanning Calorimetry

**Figure 5:**
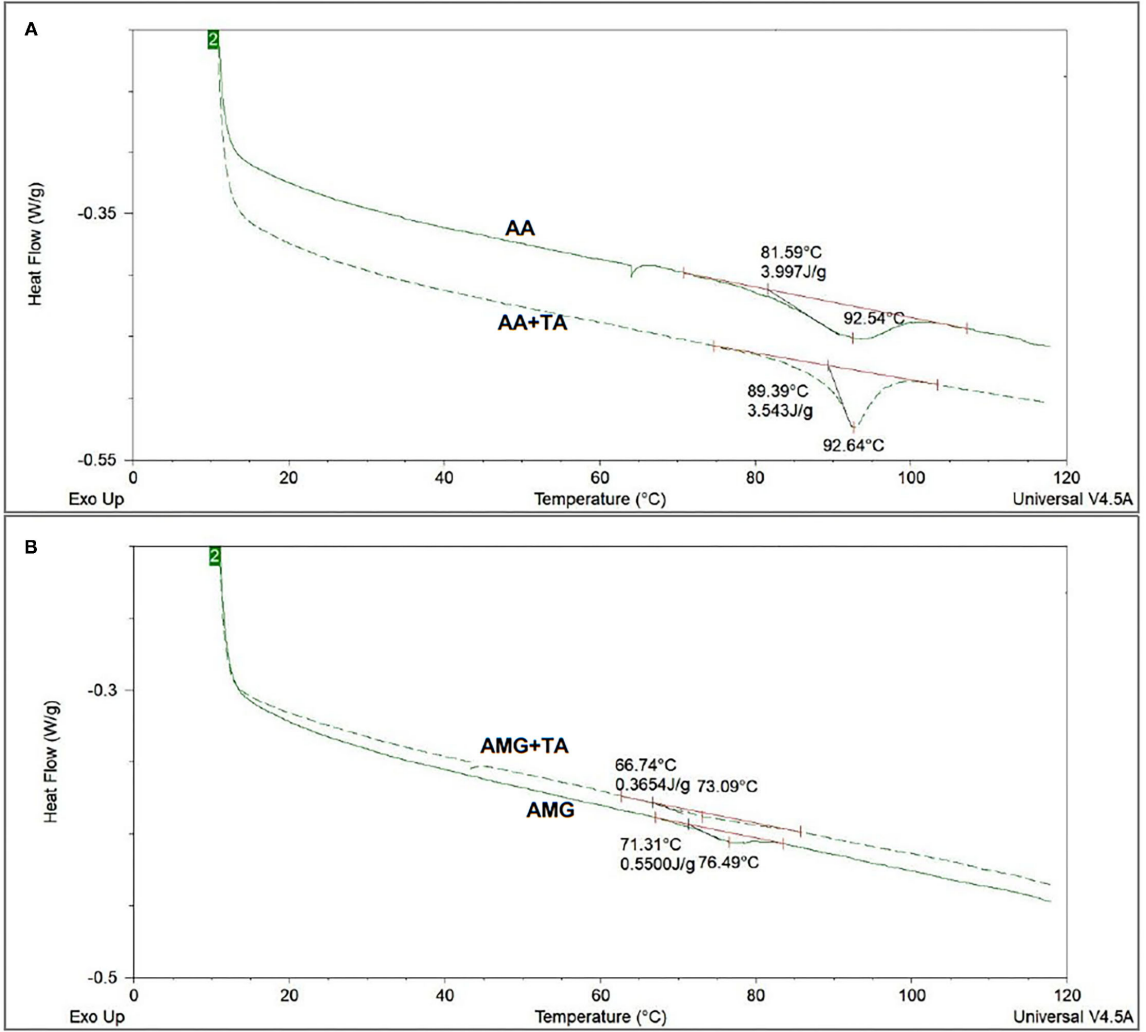
The differential scanning calorimetry (DSC) thermograms of AA **(A)** and AMG **(B)** in the absence and presence of TA, in which the denaturation temperatures (*T*_d_) and denaturation enthalpy (Δ*H*_dsc_) were obtained after integration of the heat-flow thermograms.

The DSC method was used to characterize the change in the thermal stability of enzymes caused by binding with TA (Figure 5). According to this change, the stability of spatial structures of the enzymes was speculated. It was found that the denaturation temperatures (*T*_d_) of AA and AMG were 92.54 and 76.49°C (Table 1), respectively. These results are consistent with the results from another study (38). The addition of TA maintained the thermal stability of AA but decreased that of AMG, as suggested by the unchanged *T*_d_ and Δ*H*_dsc_ of AA and by the decreased *T*_d_ and Δ*H*_dsc_ of AMG (Figure 5, Table 1).

During the process of DSC running, two steps occur for an enzyme, such as reversible unfolding, at the initial heating stage within relatively low temperature range and structural collapse caused by complete thermal denaturation at high temperatures (10, 17). The thermal stability of a protein highly depends on the compact/loose extent of its spatial structure (39, 40). Therefore, non-covalent interactions with the enzyme that may reversibly fold or unfold its spatial structure can delay or accelerate the denaturation process of the enzyme during the DSC running (16, 38, 40).

Tannic acid was suggested to reversibly unfold the spatial structure of AMG as TA accelerate the thermal denaturation of the enzyme (Figure 5), while TA did not unfold/fold AA structure. However, the peak of enthalpy change in the heat flow of AA in the presence of TA during the process of DSC running became sharper compared with the peak in the absence of TA (Figure 5). This result indicated that the binding interactions between TA and AA occurred, but did not significantly affect the structural stability of AA in general. The difference in the change of structural stability of AA and AMG in the presence of TA was likely due to the difference in the enzyme structural properties and specific amino acid sites of the enzymes that participated in binding with TA (16, 38).

#### Molecular Docking

**Figure 6:**
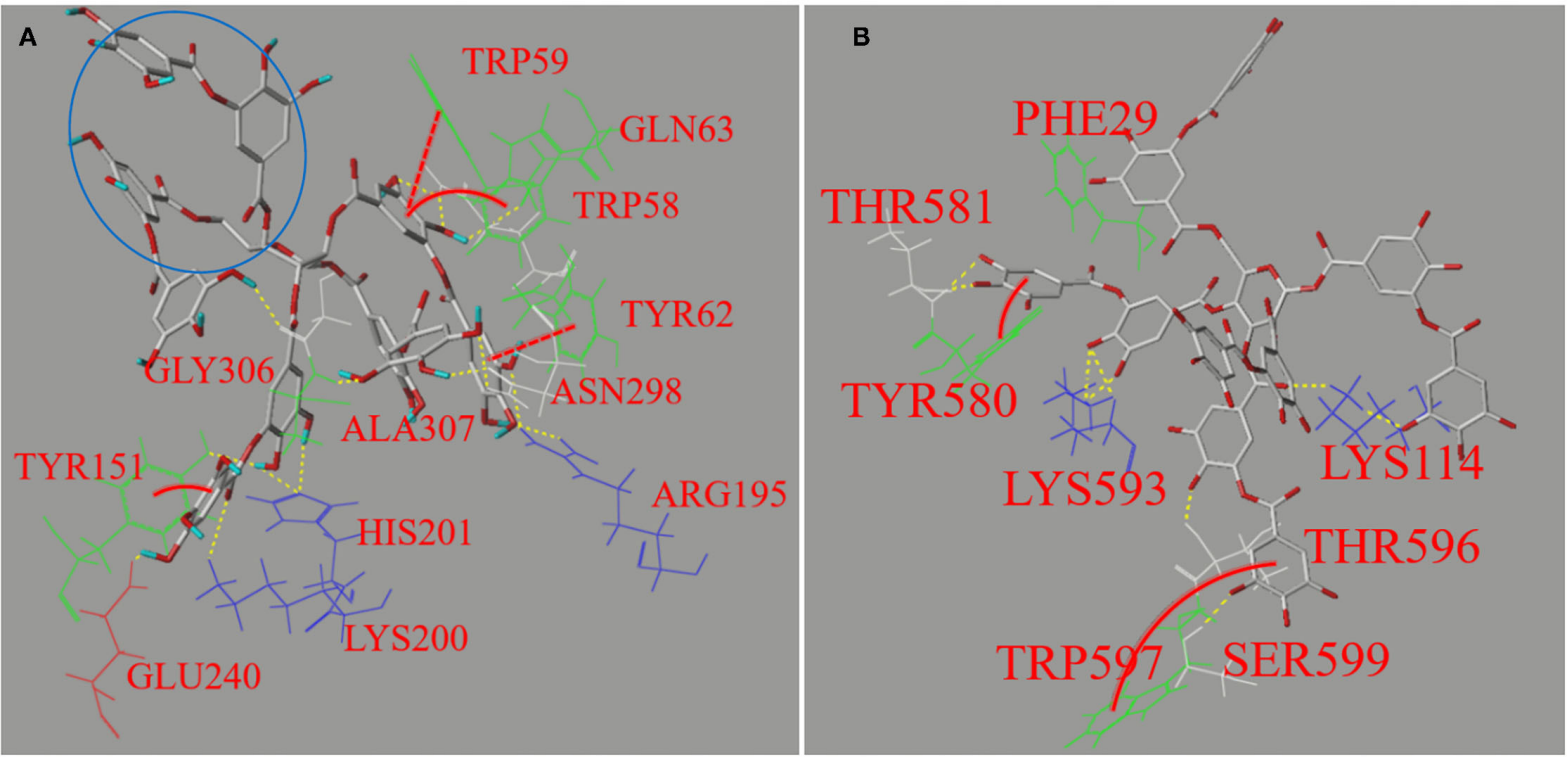
The molecular docking profiles for non-covalent binding interactions of TA with AA **(A)** and AMG **(B)**, respectively. The interaction forces between the polyphenol moieties and the amino acid residues involved hydrogen bondings (yellow dashed lines) and π-π hydrophobic forces, such as parallel conjugations (red full lines) and vertical ones (red dashed curves). Besides, the area in blue circle indicates the phenolic moieties that may not enter the active site of AA according to the applied docking procedure.

Molecular docking is an effective approach simulating the binding sites of a protein with a micromolecular ligand and the non-covalent molecular interaction forces (35). In this study, the method was applied to characterize the binding interactions between TA and the enzymes (Figure 6).

It was found that the 12 amino acid residues of AA were involved in interactions through hydrogen bondings (yellow dash lines) and π-conjugations (red lines and curves) with TA (Figure 6A), compared with 8 residues for AMG (Figure 6B), corresponding to the stronger inhibition effect on AA (Figure 2A). Four fluorescent residues (Trp^59^, Trp^58^, Tyr^62^, and Tyr^151^) of AA and two fluorescent residues (Tyr^580^ and Trp^597^) of AMG were involved in π-conjugations with the aromatic rings of TA, respectively, such as parallel conjugations (red dashed lines) and vertical conjugations (red full curves) (Figure 6). These conjugations caused the higher quenching effect of TA on the fluorescence of AA than that of AMG (Table 1). In general, the different residues and forces involved in the binding of both enzymes with TA caused the different molecular conformation of TA-amino acid residues complex at the binding sites and subsequent difference in the thermal stability of AA compared with that of AMG (Figure 5).

The simulated binding energy (*E*_b_) indicates the docking efficiency of a ligand to a protein (40). Although the binding affinity of TA to AA was higher than to AMG as suggested by ITC, the *E*_b_ value of TA for docking with AA was lower than with AMG (Table 1). This result may arise from the fact that the docking method was applied at the active pocket site of an enzyme. It is established that the docking efficiency depends on the molecular size of ligand, the size of active pocket, and position where active site is located (21, 35). The docking profile in this study suggests that these factors may favor the docking of TA to AMG because there were more adjacent moieties of TA not entering into the active pocket of AA (blue circle in Figure 6A). This result indicates that more TA molecules were required to saturate the specific binding site of AA, partially resulting in the higher stoichiometry (*n*) value for the titration of TA to AA as described in the ITC result (Figure 4). However, the molecular moieties of TA that entered the docking pocket of AA were able to form more interactions forces with the catalytic residues of AA than with that of AMG (Figure 6), causing the stronger inhibitory activity against AA (Figure 2A).

**Figure 7:**
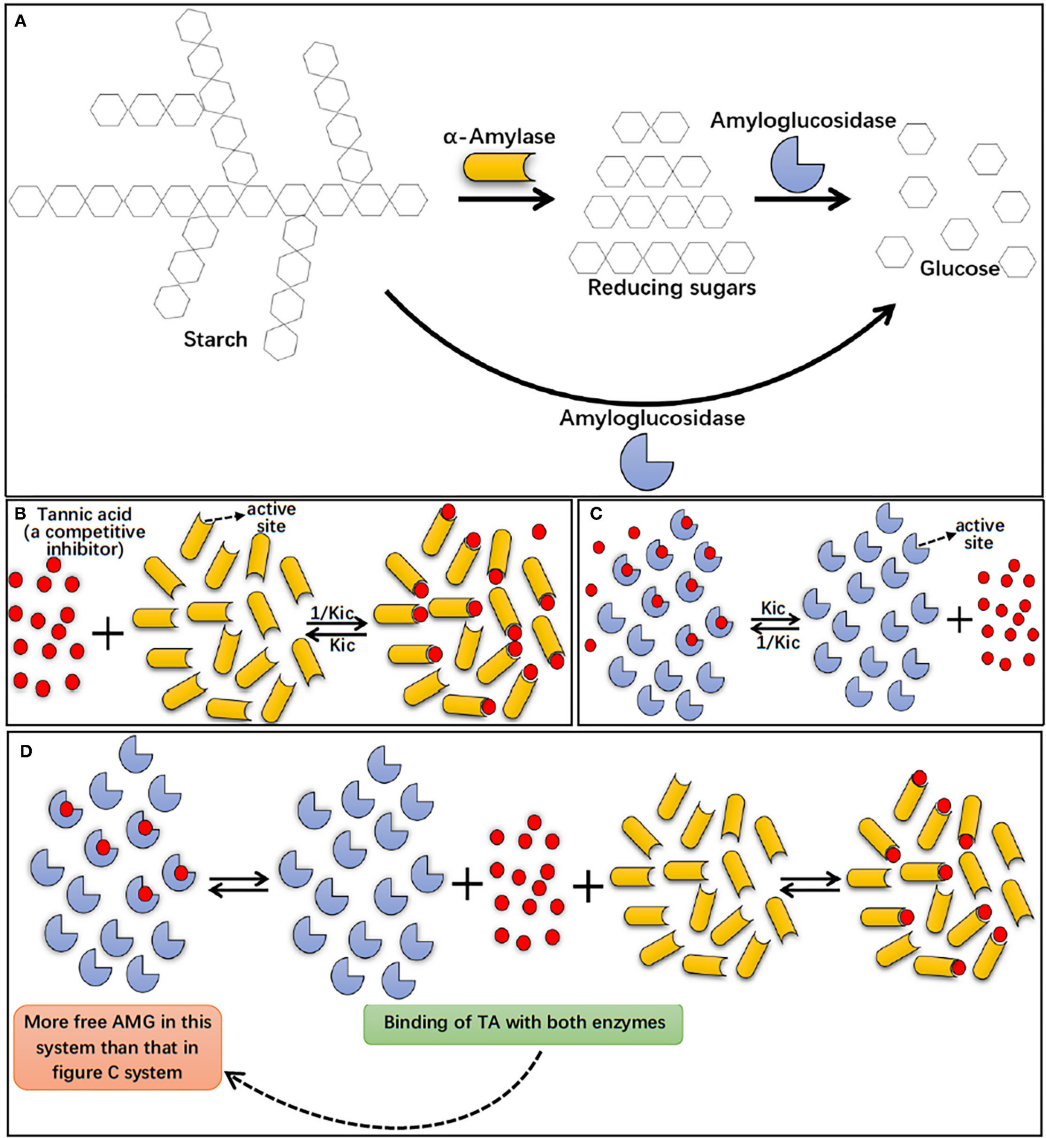
Starch digestion processes by AA and AMG, in which AA hydrolyzes starch to reducing sugars (maltose, maltotriose, maltooligosaccharides, *etc*.), while AMG is able to hydrolyze both starch and reducing sugars to glucose **(A)**. The binding interactions of TA with individual AA **(B)** and AMG **(C)**, and the binding interactions occurred in AA/AMG bi-enzyme system in the presence of TA **(D)**. Specifically, TA was a specific competitive inhibitor of both AA and AMG, indicating that the polyphenol bound with the active sites of the enzymes, competing with starch molecules (substrate). Here, *K*_ic_ indicates the dissociation constant of the TA-enzyme complex, and thus 1/*K*_ic_ suggests the association constant of TA with the enzyme. As suggested by inhibition kinetics and ITC, TA was shown with a higher binding affinity to AA than to AMG, as shown in **(B,C)** by the more TA molecules bound with AA than with AMG. In addition, in AA/AMG bi-enzyme system, binding interactions occurred both between TA and AA and between TA and AMG, this caused the more “free” AMG molecules without binding with TA in AA/AMG bi-enzyme system than that in individual AMG system (as shown in **C,D**).

In summary, the results from this study showed that AA hydrolyzed starch, producing reducing sugars, while AMG hydrolyzed both starch and reducing sugars, producing glucose (Figure 7A). The higher catalytic efficiency of AMG for more complex starchy substrates resulted in the antagonistic action between AA and AMG at the digestion phase of RDS both in the absence and presence of TA. The results of inhibition kinetics, fluorescence quenching, ITC, DSC, and molecular docking showed that TA can interact with AA and AMG, respectively, with a higher binding affinity to AA (Figures 7B,C). Therefore, the amount of AMG binding with TA in the bi-enzyme system was less than that in the individual AMG system (Figures 7C,D). As a result, the inhibition effect of TA on glucose production was weaker in the AA/AMG system than in individual AMG at the digestion phase of SDS, because glucose production only results from the catalytic action of AMG.

The above findings describe the inhibiting behaviors of a specific competitive inhibitor TA on starch digestion with individual and co-existing starch-hydrolyzing enzymes, AA and AMG, emphasizing the antagonistic effects of enzymes and inhibitor-enzyme binding interactions. These findings provide a theoretical basis for the evaluation of the inhibition effects of polyphenols on starch digestion and the potential regulating actions of polyphenols on postprandial blood glucose levels.

## Conclusion

In this study, the inhibitory effects of TA on starch digestion by AA, AMG, or AA/AMG bi-enzyme system were studied using the first-order reaction analysis. It was found that TA displayed the strongest inhibition to starch digestion with AA among the three enzyme systems. The digestion velocity of RDS with individual AMG was faster than that with AA/AMG bi-enzyme system both in the absence and presence of TA, because at this phase the antagonistic effect between AA and AMG mainly contributed to the enzyme catalyzing behaviors. However, at the digestion phase of SDS where the antagonistic effect was not obvious, the starch digestion velocity in AA/AMG bi-enzyme system with TA was faster than that in individual AMG with TA. The results of the analyses of the inhibition kinetics, fluorescence quenching, ITC, DSC, and molecular docking suggested that TA bound with AA and AMG, but the affinity was higher with AA. Therefore, the presence of AA changed the catalytic behavior of AMG both in the absence and presence of TA due to the antagonistic action of AA and AMG and the binding interactions between TA and the two enzymes.

## Data Availability Statement

The original contributions presented in the study are included in the article/Supplementary Material, further inquiries can be directed to the corresponding author.

## Author Contributions

SL: conceptualization, data curation, methodology, software, validation, and writing original draft. SZ: methodology. XY: methodology, software, and supervision. LS: project administration, writing, review and editing, project administration, and fundings acquisition. All authors contributed to the article and approved the submitted version.

## Funding

This study was supported by the National Natural Science Foundation of China (No. 32172204).

## Conflict of Interest

The authors declare that the research was conducted in the absence of any commercial or financial relationships that could be construed as a potential conflict of interest.

## Publisher's Note

All claims expressed in this article are solely those of the authors and do not necessarily represent those of their affiliated organizations, or those of the publisher, the editors and the reviewers. Any product that may be evaluated in this article, or claim that may be made by its manufacturer, is not guaranteed or endorsed by the publisher.
