# Supplementary figures and images for: α-Amylase Changed the Catalytic Behaviors of Amyloglucosidase Regarding Starch Digestion Both in the Absence and Presence of Tannic Acid
Source: Front Nutr. 2022 Apr 13;9:817039. doi: 10.3389/fnut.2022.817039 (PMC9043763; doi:10.3389/fnut.2022.817039)

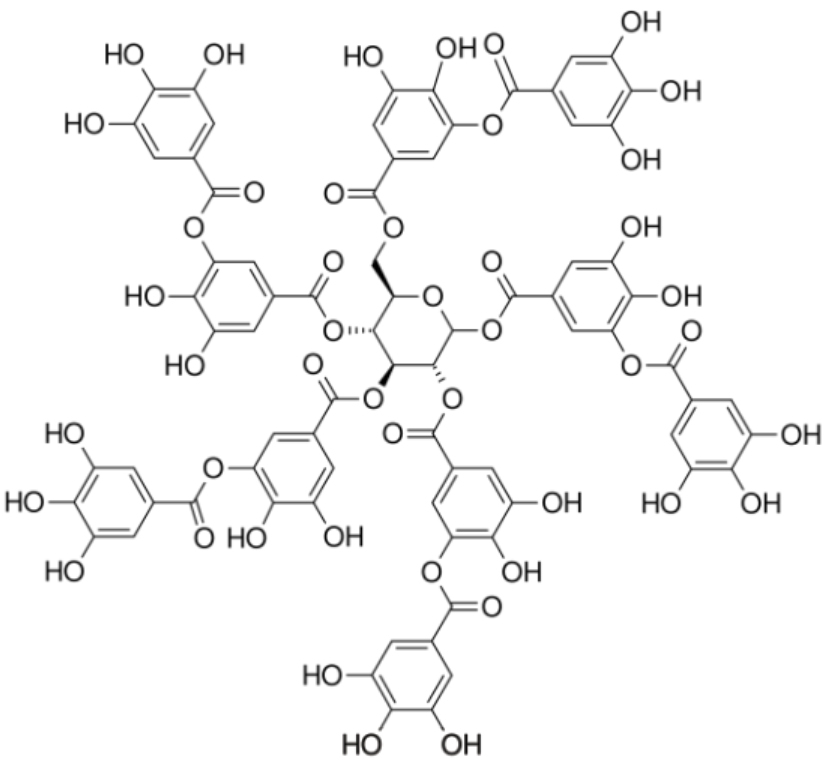

Supplement: Supplementary file 1 [file Image_1.JPEG]
